# Supplementary material for: Patterns of Intron Gain and Loss in Fungi
Source: PLoS Biol. 2004 Nov 30;2(12):e422. doi: 10.1371/journal.pbio.0020422 (PMC532390; doi:10.1371/journal.pbio.0020422)
Supplement: Table S1 — Also available at http://genes.mit.edu/NielsenEtAl/. (4.3 MB ZIP). [file pbio.0020422.st001.zip › NielsenEtAl/html/1305.html]

AN3413.1.NCU06047.1.MG09222.1.FG01509.1


```
 CLUSTAL W (1.82) Multiple Sequence Alignments - Introns Inserted


Sequence 1: NCU06047.1	265 aa
Sequence 2: FG01509.1	257 aa
Sequence 3: MG09222.1	265 aa
Sequence 4: AN3413.1	259 aa
Alignment Length: 271 aa
Number Identitical Residues: 215 aa
Alignment Score (without introns) 8383


MG09222.1 	MADAAPR---GGGFASRGGDRGGDRGRGDRGRGRG-RGRGRR~GGKSDEKEWQ~PVTKLG
NCU06047.1	MADRGTSGPRGGGFASRGD------RGGDRGRGRGGRGRGRR~GGKSEEKEWQ~PVTKLG
FG01509.1 	MADRGTS--RGGGFASRG---------GDRGRGRG-RGRGRG1RGKNEEKEWQ~PVTKLG
AN3413.1  	MADAAPR--GRGGFGSRGD------RGGDR-RGRG-RGRGRR~GGKTEEKEWQ0PVTKLG
          	*** ..     ***.***.        *** **** *****   **.:***** ******

MG09222.1 	RLVKAGKIKSMEEIYLHSLPIKEYQIVDFFLPKLKDEVMK0IKPVQKQTRAGQRTRFKAI
NCU06047.1	RLVKAGKIKSMEEIYLHSLPIKEYQIVDFFLPKLKDEVMK0IKPVQKQTRAGQRTRFKAI
FG01509.1 	RLVKAGKINSMEEIYLHSLPIKEYQIVDNFLPKLKDEVMK0IKPVQKQTRAGQRTRFKAI
AN3413.1  	RLVKAGKITSMEQIYLHSLPVKEYQIVDFFLPKLKDEVMK0IKPVQKQTRAGQRTRFKAV
          	********.***:*******:******* *********** ******************:

MG09222.1 	VIIGDSEGHVGLGIKTSKEVATAIRAAIIIAKLSVIPVRRGYWGTNLGTPHSLPVKESGK
NCU06047.1	VIIGDSEGHVGLGIKTSKEVATAIRAAIIIAKLSVIPVRRGYWGANLGLPHSLPVKESGK
FG01509.1 	VIIGDSEGHVGLGIKTSKEVATAIRAAIIIAKLSVIPVRRGYWGTNLGAVHSLPTKESGK
AN3413.1  	VVIGDSEGHIGLGIKTSKEVATAIRAAIIIAKLAVLPVRRGYWGTNLGEPHSLPVKQSAK
          	*:*******:***********************:*:********:***  ****.*:*.*

MG09222.1 	CGSVTVR0LIPAPRGTALVASPAVKRLLQLAGIEDAYTSSSGSTKTLENTLKATFSAVSN
NCU06047.1	CGSVTVR0LIPAPRGTSLVASPAVKRLLQLAGIEDAYTSSSGSTKTLENTLKATFAAVSN
FG01509.1 	CGSVTVR0LIPAPRGTGLVASPAVKRFLQLAGVEDAYTSSAGSTKTLENTLKATFVAVSN
AN3413.1  	CGSVSVR0LIPAPRGTGLVASPAVKRLLQLAGVQDAYTSSSGSTKTLENTLKATFLAVVN
          	****:** ********.*********:*****::******:************** ** *

MG09222.1 	TYGFLTPNLWKETKLIRSPLEEFADTLRDGKRY--
NCU06047.1	TYGFLTPNLWKETKLTRSPLEEFADTLREGKRYAN
FG01509.1 	TYGFLTPNLWKETKLIRSPLDEFADTLREGKRY--
AN3413.1  	TYGFLTPNLWTETKLTRSPLEEFSDVLRQGKKY--
          	**********.**** ****:**:*.**:**:*
```
